# ksRepo: a generalized platform for computational drug repositioning

**DOI:** 10.1186/s12859-016-0931-y

**Published:** 2016-02-09

**Authors:** Adam S. Brown, Sek Won Kong, Isaac S. Kohane, Chirag J. Patel

**Affiliations:** Department of Biomedical Informatics, Harvard Medical School, Boston, MA 02115 USA; Boston Children’s Hospital, Boston, MA 02115 USA

**Keywords:** Repositioning, Drug discovery, Prostate cancer, Gene expression

## Abstract

**Background:**

Repositioning approved drug and small molecules in novel therapeutic areas is of key interest to the pharmaceutical industry. A number of promising computational techniques have been developed to aid in repositioning, however, the majority of available methodologies require highly specific data inputs that preclude the use of many datasets and databases. There is a clear unmet need for a generalized methodology that enables the integration of multiple types of both gene expression data and database schema.

**Results:**

ksRepo eliminates the need for a single microarray platform as input and allows for the use of a variety of drug and chemical exposure databases. We tested ksRepo’s performance on a set of five prostate cancer datasets using the Comparative Toxicogenomics Database (CTD) as our database of gene-compound interactions. ksRepo successfully predicted significance for five frontline prostate cancer therapies, representing a significant enrichment from over 7000 CTD compounds, and achieved specificity similar to other repositioning methods.

**Conclusions:**

We present ksRepo, which enables investigators to use any data inputs for computational drug repositioning. ksRepo is implemented in a series of four functions in the R statistical environment under a BSD3 license. Source code is freely available at http://github.com/adam-sam-brown/ksRepo. A vignette is provided to aid users in performing ksRepo analysis.

## Background

Repositioning of previously approved drugs is a promising methodology because it reduces the cost and duration of the drug development pipeline and reduces the likelihood of adverse events [1–4]. High-throughput repositioning efforts are especially appealing given their ability to yield many potential development opportunities [5–7]. A major goal in repositioning is the development of *in silico* tools that reduce the number of potential candidate molecules to be screened while also suggesting unlikely and novel possibilities. To this end, a number of groups have developed computational approaches that display high degrees of both sensitivity and specificity [8]. Many successful computational repositioning methodologies have relied on comparing individual disease RNA-level expression profiles to large databases of pre-generated multi-drug exposure profiles or known gene-drug interactions [9–12]. Unfortunately, the majority of these methodologies are hindered by their need for specific data types and formats, including requirements for detailed genomic or phenotypic annotations [9, 10], expression levels from a single microarray platform [12], and pre-determined databases of drug-gene interactions [13]. These limitations prevent investigators from utilizing newer profiling technologies, such as RNA-seq, and from utilizing alternative or proprietary compound exposure profiles. Despite these drawbacks, numerous successes using these techniques, including the highly cited Connectivity Map (Broad), suggest the utility of a pipeline capable of surpassing these hindrances [12, 14–17].

**Fig. 1:**
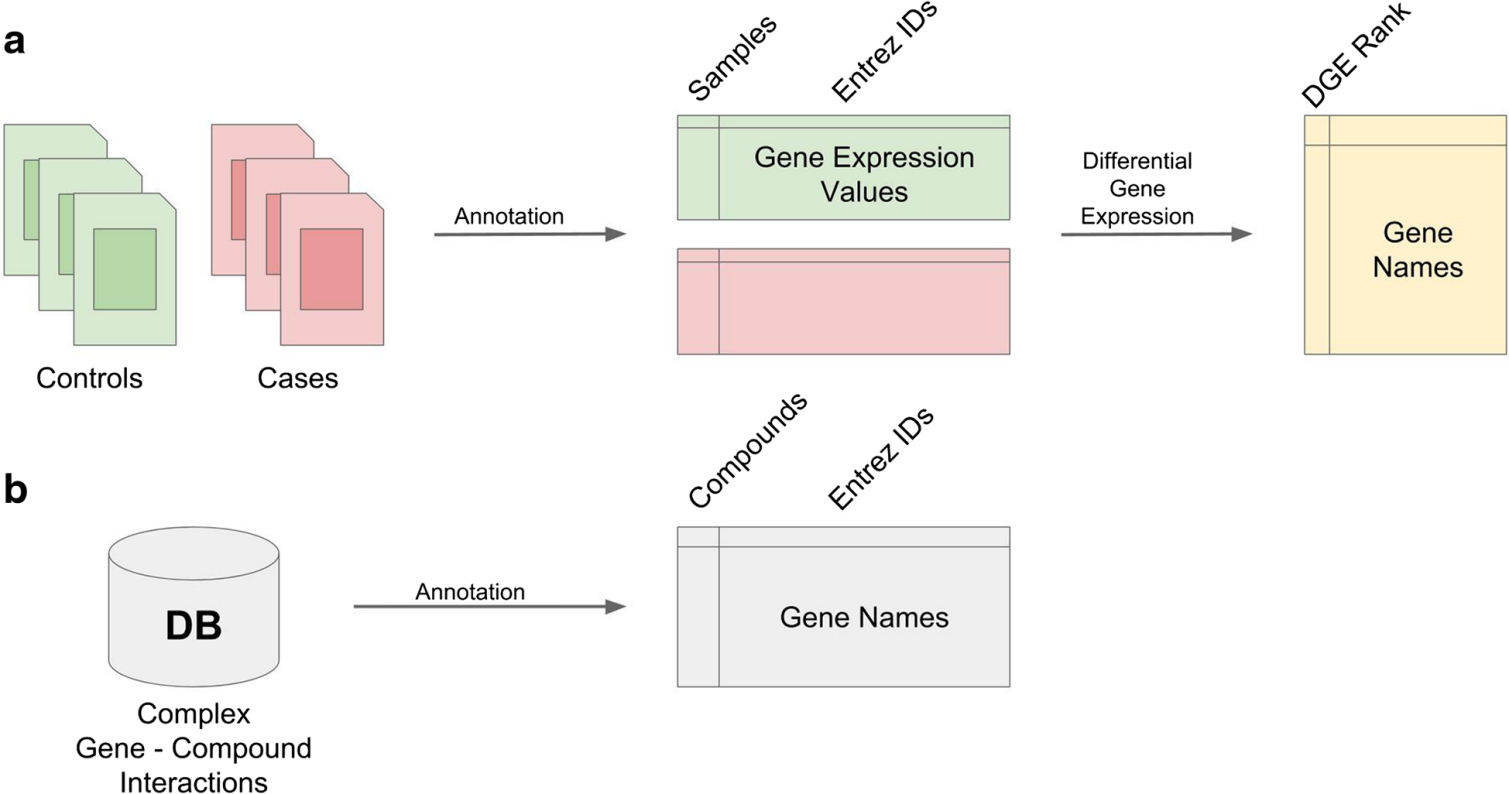
*ksRepo* can use *any* input data types. **a** Preparation of arbitrary expression data is accomplished by conversion to Entrez ID using manufacturer or online annotation tools followed by the user’s preferred algorithm for determining differential gene expression. **b** Preparation of an arbitrary database only required conversion to Entrez ID, which is often provided by the database directly

To address these limitations, a universally applicable computational repositioning tool should have flexibility in the types of data sets and databases that can be used. Specifically, we envision such a tool having 1) the ability to interrogate *any* case/control disease study-derived expression profile, 2) the ability to use *any* compound database, including those with limited numbers of gene-drug interactions, and 3) an extensible, open-source distribution. Here, we propose a generalized tool for computational repositioning that builds on the successes of previous expression-based repositioning tools while allowing greater flexibility for the investigator called *ksRepo*. Our methodology modifies the Kolmogorov-Smirnov (KS) enrichment approach used by the Broad Connectivity Map to enable the use of any expression-level disease study with any database containing, at minimum, gene-drug interactions from any source (with or without information about the directionality of association) [12, 13]. The only requirement of our methodology is that there is a common identifier system to which the information from both the disease and exposure databases can be converted (see Fig. 1). Unlike many popular repositioning tools (e.g. [9–13]), we provide source code for *ksRepo* that enables investigators to extend our methodology as new datatypes become available. We demonstrated our methodology using five independent freely available Prostate Cancer datasets [18–22] downloaded from the Gene Expression Omnibus (GEO) [23] and the open-source gene-drug interaction database, the Comparative Toxicogenomics Database (CTD) [24].

## Methods

### GEO dataset processing

All GEO datasets were accessed through the NCBI GEO portal and analyzed using the integrated GEO2R tool [23]. As input for GEO2R, we classified each sample within a GEO series as either normal tissue or tumor tissue. GEO2R provides a list of all probes (and corresponding gene aliases) ranked according to their degree of differential expression. We imported all of the results from GEO2R into R [25] and converted all gene aliases into EntrezGene Identifiers using the org.Hs.eg.db R package [26]. The prostate cancer datasets used in this study are GSE3868, GSE12348, GSE45016, GSE55945, and GSE6919 [18–22]. The five prostate cancer datasets were chosen on the basis of three criteria: (1) the expression profiles were derived from primary prostate cancer cells, as opposed to cell lines or short-term cultures, (2) there were healthy prostate tissue controls included in the study, and (3) tissue samples were from fresh-frozen biopsies, and not preserved (e.g. by FFPE).

### CTD database construction

To generate a ksRepo-compatible database, we first downloaded the entirety of the CTD and imported the database into R (downloaded February, 2014). The CTD contains manually curated compound-gene interactions collected from the primary literature by trained experts [24]. We filtered the full database for literature-supported interactions between compounds and human genes or gene products (e.g. transcripts, proteins, or peptides). Following filtering, the resulting database contained interactions between 7170 compounds and 18,768 unique human genes. Of these 7170 compounds, 1660 are drugs approved by the FDA. A script for converting the downloadable files from CTD to a ksRepo compatible format is available in the ksRepo GitHub repository (CTDget.R script).

### Kolmogorov-Smirnov enrichment score calculations

Our modified method is analogous to an “inverse” version of the Connectivity Map implementation in that we compare a single **instance** (complete gene expression profile) to a number of **signatures** (short compound-gene interaction lists) rather than comparing a single **signature** to a number of **instances**. In addition, we focus on interaction without directionality to accommodate compound exposure databases with no regulatory component or conflicting regulatory information. In addition, we consider all genes in the ranked **instance** gene list regardless of significance to ensure overlap between the **instance** and **signatures**. KS enrichment scores for our method are calculated as follows.

Let *n* be the number of genes in the **instance** and *t* be the number of genes in a given **signature**. Order all *n* genes in the **instance** by their differential expression. Construct a vector *V* of the position (∈ {1,…,*n*}) of each **signature** gene in the **instance** ordered gene list and sort these components in ascending order such that *V(j)* is the position of gene *j*, where *j* ∈ {1,…,*t*}. Calculate the following values:

1 $$ a=\underset{j= 1}{\overset{t}{max}}\left[\frac{j}{t}-\frac{V(j)}{n}\right] $$

2 $$ b=\underset{j= 1}{\overset{t}{max}}\left[\frac{V(j)}{n}-\frac{\left(j- 1\right)}{t}\right] $$

and set *KS* = *a* if *a* > *b*. Else set *KS* = −*b*.

Both *a* and *b* quantify differences in the expected distribution of gene ranks (∈ {1,…,*n*}) and the observed sample of ranks in the **signature**. The value *a* ∈ U(0,1) and scales inversely with the *mean***signature** rank (mean *V(j)*), with deviations proportional to the *standard deviation* of **signature** rank; the value *b* is the inverse of *a*. If *a* > *b*, then the mean **signature** rank is low, corresponding to enrichment, and we assign *a* as the *KS* score. If *b* > *a*, we assign -*b* as the *KS* score. In this way, **signatures** with highly enriched gene sets are assigned highly positive *KS* scores, while **signatures** with unenriched or inversely enriched (e.g. very high *mean***signature** rank) are assigned *KS* scores near zero or negative *KS* scores respectively.

Because our *KS* test statistic has no empirical distribution, we calculated significance by bootstrapping as follows. Construct a vector *L* of the number of genes in each **signature**. For each unique *ℓ* in *L*, generate 10,000 independent resamples of the **instance** gene list of length *ℓ* and calculate *KS* scores for each resample. For each **signature**, compare the observed **signature***KS* score to the corresponding resample with the same number of genes. Set the *p value* of that signature as the proportion of resample *KS* scores that exceed the **signature***KS* score and FDR adjust to correct for multiple hypothesis testing [27].

### ksRepo implementation

**Fig. 2:**
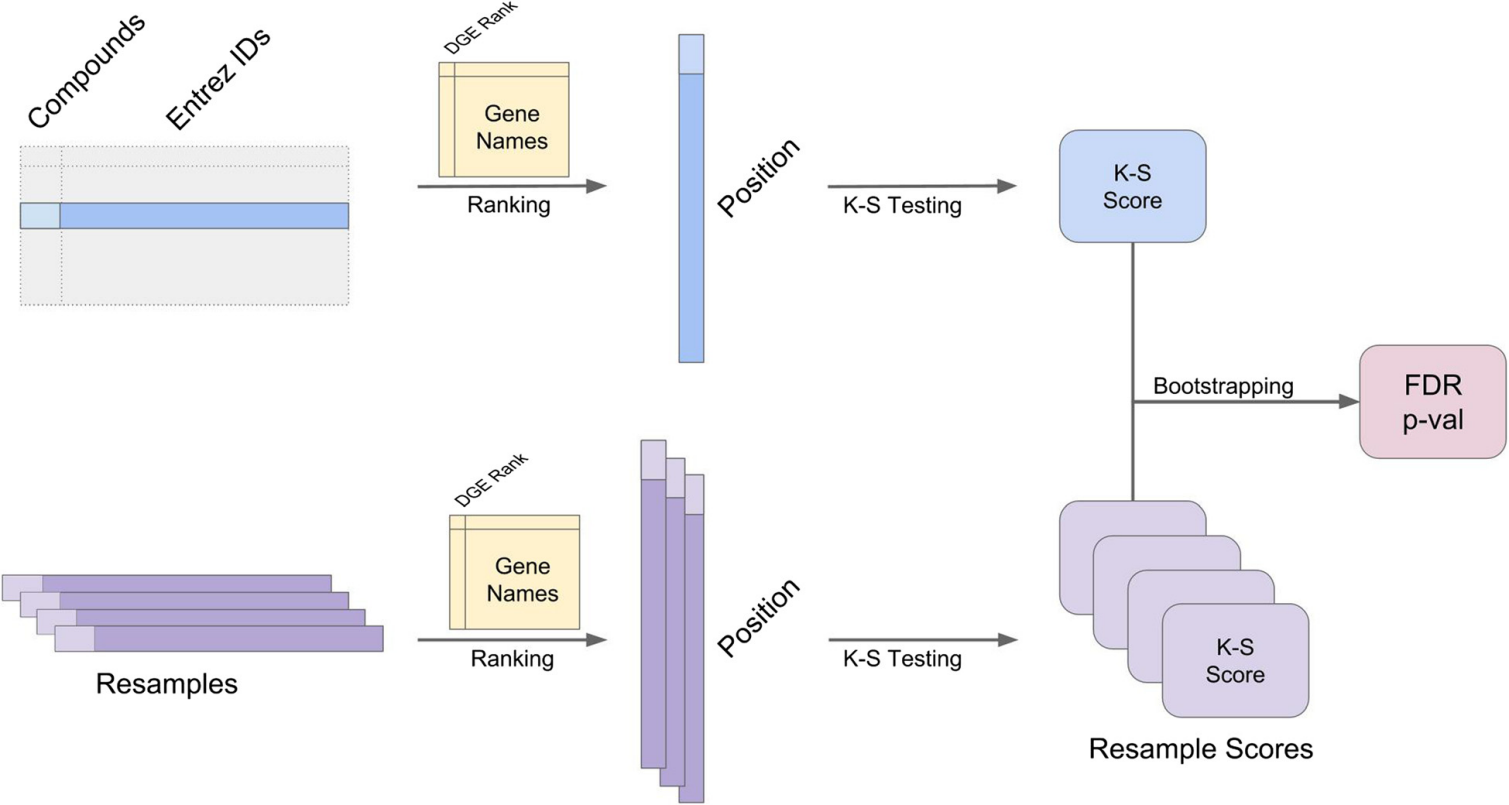
*ksRepo* employs a generalized K-S enrichment test and bootstrapping to determine candidate repositioning targets. See Methods for a detailed description

We implemented ksRepo testing in R as a series of four functions. The core testing function, *ks_single*, performs KS enrichment testing between the ranked **instance** gene list and one unranked **signature** gene list (see Fig. 2). Bootstrapped *P*-value calculation is accomplished by the functions *boot_ks* and *boot_p*. The final function included in the implementation, *repo*, is a wrapper function which calls the other three functions and formats the output. All four functions are available for non-commercial use from GitHub (http://github.com/adam-sam-brown/ksRepo). In addition, we provide a comprehensive vignette that demonstrates the use of ksRepo using one of the prostate cancer datasets, GSE6919, described above.

## Results and discussion

We implemented ksRepo, an expression-based, generalized tool for computational drug repositioning. *ksRepo* avoids the requirements of currently available methods for specific data inputs [9–13]. Our methodology is capable of utilizing any pair of disease expression dataset and compound exposure database with the simple constraint that they be mappable to a single, common identifier system. Once an investigator has chosen the two inputs, our method straightforwardly expands the methods of Lamb and colleagues [12] to allow for varying numbers and types of gene-compound associations in the exposure database. We then compute K-S enrichment scores for each compound and report bootstrapped and FDR-corrected *p*-values for ease of interpretation.

To demonstrate ksRepo’s applicability, we applied our method to five independent prostate cancer datasets (GSE3868, GSE12348, GSE45016, GSE55945, and GSE6919) from three distinct microarray platforms, and attempted to detect signal from FDA-approved prostate cancer therapies from the CTD. The CTD reports expert curated gene-compound interactions from the primary literature. Unlike currently available methods (e.g. [9–13]), in which a full profile is necessary, ksRepo is able to analyze the CTD and databases like it. We also note that the use of three distinct microarray platforms precludes the use of some methods (e.g. [12, 13]), but is possible with ksRepo.

We first identified all FDA-approved prostate cancer therapies using DrugBank [28] and then determined that out of 11 small-molecule therapies, seven (Bicalutamide, Nilutamide, Leuprolide, Zoledronic Acid, Docetaxel, Aminoglutethimide, and Estropipate) were also included in the CTD. We then applied ksRepo to the five GEO prostate cancer datasets and determined the FDR-corrected *p*-values for each of the seven annotated therapies in the CTD. ksRepo predicted on average approximately 300 compounds (median 319 compounds, FDR-corrected *p*-value <0.05) corresponding to a specificity around 5 %, which is similar to reported specificities for other repositioning strategies [9–13].

For each of the five prostate cancer datasets we were able to detect significance for between one and three FDA-approved therapies at a FDR-corrected *p*-value less than 0.05. In each case, this represented a significant enrichment for approved therapies (Hypergeometric Test, *p* < 0.027, expected number of drugs μ = 0.029). Among compounds, significant prostate cancer therapies ranked on average in the 3.5th percentile and of the seven therapies, five were significant for at least one of the five datasets. We did not detect significance for two therapies, Aminoglutethimide and Estropipate; we hypothesize that due to the nature of the microarray datasets we included (tissue from primary, non-metastatic tumors), it is unlikely that we would detect secondary hormone modulatory treatments, which are typically used in treatment refractory patients with metastases [29].

These results suggest that ksRepo is a generalized methodology for computational drug repositioning. Even after intentionally reducing the information content presented to our methodology by using a database with a modest number of gene interactors by compound (as annotated in the CTD), we were still able to recover many of the FDA-approved drugs for prostate cancer. In addition, we have enabled the use of any microarray platform as input, bypassing an impediment to using a popular repositioning tool, the Broad Connectivity Map. By allowing investigators to choose any expression study and drug exposure database we hope to spur the analysis of as-yet unexplored diseases and databases. Furthermore, because ksRepo is flexible and generalized, we hope to apply it to a variety of future projects, including the incorporation of other exposure databases such as Drugbank [28] and PharmGKB [30], as well as new input types such as mRNA-seq and epigenomic information.

## Conclusions

Here, we have described ksRepo, a generalized, expression-level tool for computational drug repositioning. Our implementation enables investigators to choose any case/control disease study and exposure database to suit their experimental needs. To validate our method, we applied ksRepo to five distinct prostate cancer datasets and the Comparative Toxicogenomics Database (CTD) and ksRepo successfully detected significance for a majority of FDA-approved prostate cancer therapies and significantly enriched for these compounds from the CTD. Our methodology is implemented in an open-source GitHub repository for free use. Future work with ksRepo will focus on exploring as-yet under utilized databases and the possibility of incorporating novel expression and genomic information.

### Availability and requirements

Project Name: ksRepo

Project Home Page: http://github.com/adam-sam-brown/ksRepo

Operating System: Platform Independent

Programming Language: R

License: BSD-3

## Abbreviations

FDA: Food and Drug Administration of the United States of America
CTD: comparative toxicology database
K-S: Kolmogorov-Smirnov
GEO: gene expression omnibus
FDR: false discovery rate
